# Case Report: Treatment with magnetic nanoparticle-based hyperthermia stabilizes metastatic disease in stage IV primary liver cancer patients

**DOI:** 10.3389/fonc.2025.1742249

**Published:** 2026-01-15

**Authors:** Sarah Kraus, Shir Arbib, Hila Ventura-Bixenshpaner, Pazit Rukenstein, Meirav Hirsh-Kovacs, Boaz Shalev, Moshe Eltanani, Udi Ron, Olga Gumina, Ofer Shalev, Jeff Geschwind

**Affiliations:** 1 New Phase Ltd., Petah Tikva, Israel; 2 Department of Radiology, Meir Medical Centre, Kfar Saba, Israel; 3 Oncology, Image-Guided Therapy, and Imaging Core Lab, North American Science Associates (NAMSA), New York, NY, United States

**Keywords:** alternating magnetic field, duty cycle modulation, iron oxide nanoparticles, magnetic nanoparticle-based hyperthermia, primary liver cancer

## Abstract

The First-in-Human clinical trial of a novel magnetic nanoparticle-based hyperthermia therapy consisting of iron oxide multicore encapsulated nanoparticles, termed Sarah Nanoparticles (SaNP), administered intravenously before being exposed to alternating magnetic field (AMF) irradiation began enrolment. We report the preliminary results on two patients with advanced primary liver cancer; a 75-year-old man with advanced stage hepatocellular carcinoma and a 69-year-old woman with metastatic cholangiocarcinoma treated with a SaNP dose of 1.8 mg/kg followed by pulsed AMF irradiation for a total duration of 15 min. Toxicity assessment was performed using standard criteria for the grading of adverse events during treatment and after a follow-up period of 30 days. Tumor response was evaluated by the Response Evaluation Criteria in Solid Tumors (RECIST) 1.1. Both patients tolerated the procedure well without any significant clinical or laboratory toxicities. Magnetic resonance imaging (MRI) analysis revealed accumulation of the SaNP in the tumors followed by clearance from the liver and spleen on T2*-weighted MR imaging. Tumor response as assessed by RECIST 1.1 demonstrated stable disease at the follow-up timepoint. Preliminary analysis of this First-in-Human trial of magnetic nanoparticle-based hyperthermia treatment for patients with advanced cancer demonstrated encouraging results both in terms of efficacy by halting disease progression and toxicity, where no significant side effects occurred.

## Introduction

Magnetic nanoparticle-based hyperthermia is a promising non-invasive nanotechnology for targeted cancer therapy that exploits the activation of magnetic nanoparticles by an external alternating magnetic field (AMF), thereby heating solid tumors without damaging healthy surrounding tissues (1–3), because cancer cells are far more sensitive to thermal deposition than healthy cells as a result of their lower heat dissipation and reduced heat capacity (4).

Sarah Nanotechnology System is a novel therapy consisting of Sarah Nanoparticles (SaNP) and an Electromagnetic Induction System (EIS), designed to treat metastatic solid tumors through the transfer of thermal energy to cancer cells at a sub-ablative temperature of up to 50 ± 3°C. The external AMF generates SaNP-induced heat in the tumor area, leading to cellular stress and cancer cell death.

These SaNP contain a multicore of encapsulated iron oxide (IO) superparamagnetic nanoparticles, a phase change material that is configured to absorb latent heat of fusion by undergoing a change in phase-state important for thermal energy storage, and a nontoxic, non-immunogenic outer layer made of polyethylene glycol (5). The nanoparticles are administered intravenously (IV) to the patient in an aqueous colloidal solution and accumulate within the tumor area through enhanced permeability and retention (EPR) effect. Following SaNP deposition and accumulation within tumors, regional AMF is applied on the torso area (chest to pelvis) with the EIS at a frequency of 290 ± 10% kHz and field strength at an amplitude modulation mode, where AMF irradiation is applied in pulses (1–10 mT) with a 50% duty cycle modulation.

Initial results on two patients who were irradiated using AMF at a constant field strength after SaNP administration, demonstrated the safety profile of this new therapeutic approach without any evidence of significant toxicities (6, 7).

Extensive pre-clinical research demonstrated that SaNP is biocompatible and chemically stable. Furthermore, SaNP administration followed by AMF exposure was highly effective in reducing tumor burden in a murine metastatic cancer model, without causing any significant toxicities (8). Biodistribution studies in swine revealed that SaNPs were cleared from the body in a dose- and time-dependent manner through the hepatobiliary route (9).

After having launched the First-in-Human clinical trial of Sarah Nanotechnology System in patients with advanced cancer, we report on two patients with advanced primary liver cancer who were treated with this new therapy. Both patients, one with hepatocellular carcinoma (HCC) and the other with cholangiocarcinoma responded well to the treatment by imaging (stable disease) in the short term without incurring any toxicities.

## Case description

The cases presented herein are part of a First-in-Human ongoing trial performed following the ethical principles of the Institutional Review Board (IRB), the local Ministry of Health (MoH), and Good Clinical Practice (GCP) guidelines. The patients signed a written informed consent prior to enrolling into the study.

The purpose of the study is to assess safety and initial signs of treatment efficacy. Toxicity evaluation includes monitoring the incidence and severity of adverse events (AEs) and serious adverse events (SAEs), characterized and graded by the Common Terminology Criteria for Adverse Events (CTCAE) 5.0. Adverse events are assessed after treatment and recorded on an electronic case report form (eCRF). Disease progression is assessed by computed tomography (CT) imaging of target and non-target lesions at 30 days post treatment, using RECIST 1.1. Inclusion and exclusion criteria are as previously described (7, 8).

Before, during and after treatment, the patients were monitored for vital signs that included ECG, blood pressure, oxygen saturation, heart rate, body surface, and oral temperature monitoring. Blood and urine samples were collected for clinical pathology and urinalysis at the following timepoints: baseline, 1 hour, and 30 days after AMF irradiation. Blood testing included complete hematology, coagulation, and clinical chemistry panels. After the treatment was completed, the patients answered a questionnaire on pain and discomfort.

SaNP accumulation in the liver, spleen, and selected tumors, and SaNP clearance from the liver and spleen were evaluated by MR imaging (Philips Healthcare 3T Ingenia MR scanner) at baseline, 4hrs. post injection, and 30 days after treatment using a T2*-weighted sequence. All MR image analyses were performed using MATLAB^®^ R2020b (MathWorks, Inc., MA, USA). The analysis included motion correction between the T2* weighted images of each MRI scan, performed using the SPM12 software (The Wellcome Centre for Human Neuroimaging, UCL, London, UK), and a spatial registration made between the different timepoints. Regions of interest in the liver, spleen, tumors, and a reference area were outlined manually. Average and standard deviation T2* from the T2* maps and from each of the T2*-weighted images were extracted and normalized to the average value of the reference area in the same slice/s.

Patient 1, a 75-year-old male with a history of hypercholesteremia, hypertension, and diabetes, presented with Barcelona Clinic Liver Cancer (BCLC) stage C HCC and evidence of metastatic lesions in the lungs, liver, and adrenal gland totaling 174 mm, was referred to the trial after exhausting all other treatment options. Previous anticancer therapy included a combination of pembrolizumab and lenvatinib for a period of ~10 months, until disease progression.

Patient 2, a 69-year-old female with a history of breast cancer, hypertension, hyperlipidemia, and hypothyroidism, presented with stage IV intrahepatic cholangiocarcinoma and multiple liver lesions, totaling 96 mm. The patient’s previous treatments included a combination of cisplatin, gemcitabine and durvalumab, for a period of ~14 months until disease progression, after which she was referred to the trial. The patient was receiving hormonal therapy (letrozole) due to her breast cancer history at the time of recruitment into the trial.

Both patients were treated with the SaNP at a dose of 1.8 mg/kg, followed by pulsed AMF irradiation (1–10 mT) within the torso area, for a total duration of 15 min. divided into 3 intervals of 5 min. each. SaNP dose was calculated based on the No-Observed-Adverse-Effect-Level (NOAEL) approach according to the FDA guidance document (10), based on the weight of the patients on the day of treatment, and SaNP batch IO concentrations. The NOAEL is the highest dose tested in an animal species that does not produce adverse effects and corresponds to a clinical SaNP dose, extrapolated from mice to swine to the human equivalent dose (HED). The first clinical dose started at 10% of the 100% HED, which was 0.12 mg/kg, after applying a safety factor of 10, according to the FDA guideline. Subsequent doses were 0.36, 0.6, 0.84, 1.2, and 1.8 mg/kg as in the current report.

The main irradiated target area included the liver which was positioned at the isocenter of the radiofrequency induction coil generating the magnetic field. As part of the treatment, about 4hrs. after SaNP administration and before AMF irradiation, the patients received an antacid (Maalox oral suspension) to neutralize gastric acid, minimize electric conductivity, and avoid gastric heating. During AMF irradiation, the patients were sedated with morphine for pain control and wrapped around the torso with a chiller-connected circulating-water cooling blanket system (CBS) to cool the patient’s irradiated area. Surface temperatures were constantly monitored throughout the procedure by infrared fiber optic temperature probes located at specific points throughout the torso as previously described (6, 7). Oral temperature was recorded before and after each AMF irradiation cycle.

The procedures were well tolerated without any laboratory or clinical findings. The accumulation and time-dependence clearance of the SaNP from the tumors as well as the liver and spleen, both key parts of the mononuclear phagocyte system (MPS), were evaluated by MRI, based on the superparamagnetic properties of the IO-containing SaNP leading to a strong effect on proton relaxation times (11). Thus, IO nanoparticles cause signal loss, appearing as a darker region on T2*-sensitive MR imaging, indicative of SaNP accumulation.

**Figure 1:**
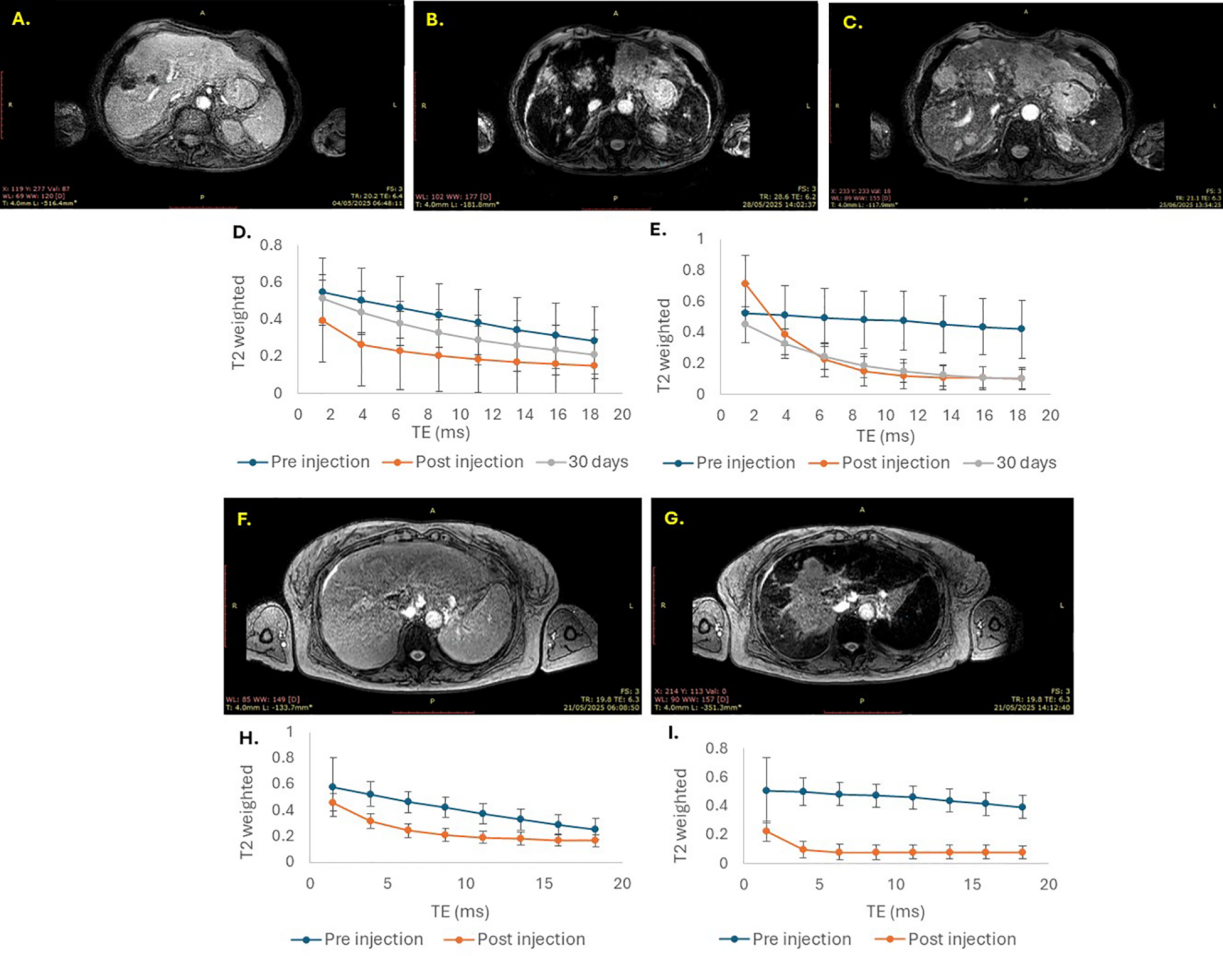
MRI analysis of SaNP accumulation in and clearance from the liver and spleen. MR imaging was obtained using a T2* imaging sequence. MR imaging of patient 1 at pre-injection **(A)**, post-injection **(B)**, and after 30 days **(C)**; T2*-weighed signals of liver **(D)** and spleen **(E)**. MR imaging of patient 2 at pre-injection **(F)**, and post-injection **(G)**; T2*-weighed signals of liver **(H)** and spleen **(I)**. Blue line – pre-injection (baseline); Orange line – post-injection (~4hrs.) and grey line – 30 days after treatment. Echo time (TE) was measured in milliseconds (ms).

For patient 1, MR imaging (**Figures 1A–E**) revealed SaNP accumulation in both the liver and spleen, as demonstrated by the low signal intensity (i.e., dark) on the post-injection MR scan. At the 30-day follow-up imaging timepoint, minimal and partial clearance of the SaNP was noted from the spleen and liver, respectively, perhaps owing to the reduced liver function resulting from the high tumor burden in the liver of this patient.

Patient 2 did not undergo MR imaging at the follow-up timepoint due to technical issues with the MR scanner and therefore, SaNP clearance analysis was not conducted. However, accumulation of the SaNP in the liver and spleen was visible on MR imaging as areas of signal loss (**Figures 1F–I**). Of note, because of disease stabilization according to RECIST 1.1, patient 2 was eligible to receive a second treatment session at the same dosage, and MR imaging was conducted one month after the second treatment, showing complete clearance from the liver with values returning to baseline levels, and partial clearance from the spleen similarly to patient 1 (data not shown).

**Figure 2:**
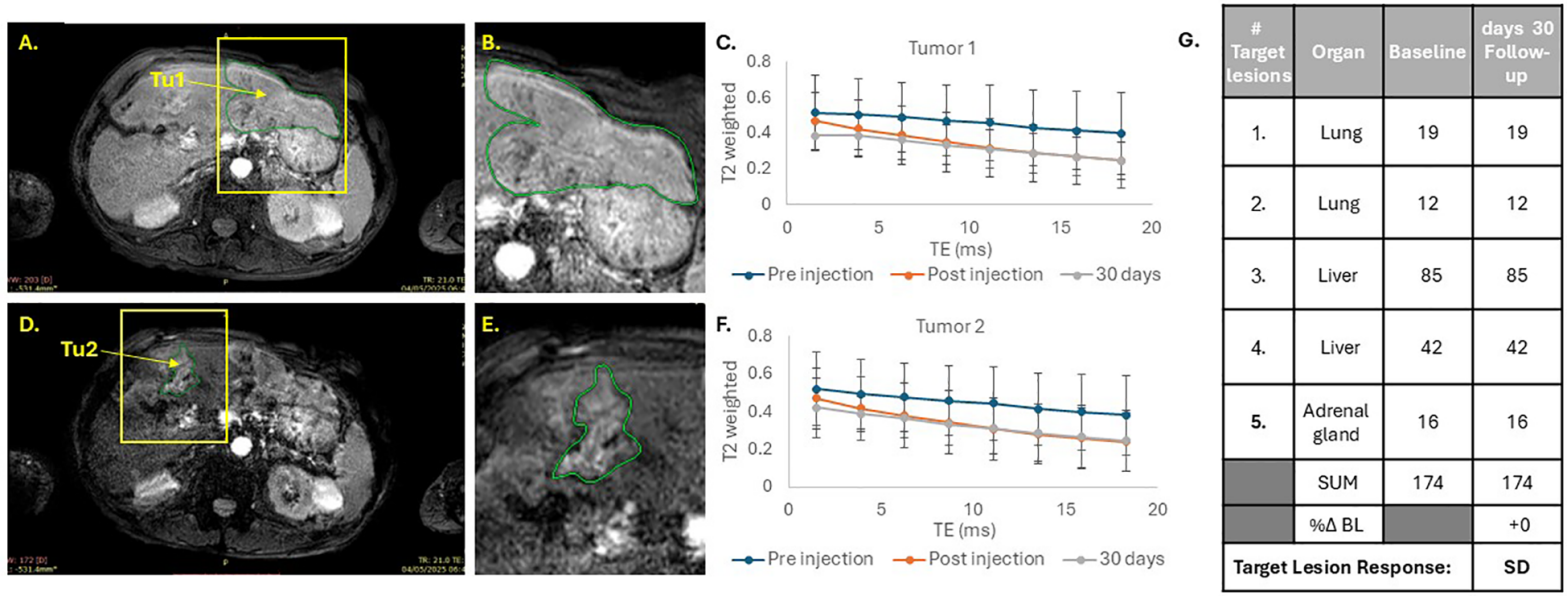
MRI analysis of SaNP accumulation in tumors of patient 1. T2*-weighted MR images **(A, B, D, E)**. Yellow arrows indicate tumor locations. Tumors are marked in green. T2*-weighed signals of tumor Tu1 **(C)** and tumor Tu2 **(F)**. Blue line – pre-injection (baseline); Orange line – post-injection (~4hrs.) and grey line – 30 days after treatment. Echo time (TE) was measured in milliseconds (ms). CT assessments and RECIST 1.1 evaluation **(G)**.

Although patient 1 presented with several tumors in the liver and lungs, SaNP only accumulated in two liver tumors that were both relatively hyperenhancing on pre-treatment imaging (labeled as tumor 1- Tu1 and tumor 2- Tu2). No accumulation of SaNP was detected in other lesions (**Figure 2**). Tumor response assessed by CT imaging 30 days post treatment revealed no measurable changes in the size of the target lesions, with a %Δ BL of 0%, indicating stable disease according to RECIST 1.1.

**Figure 3:**
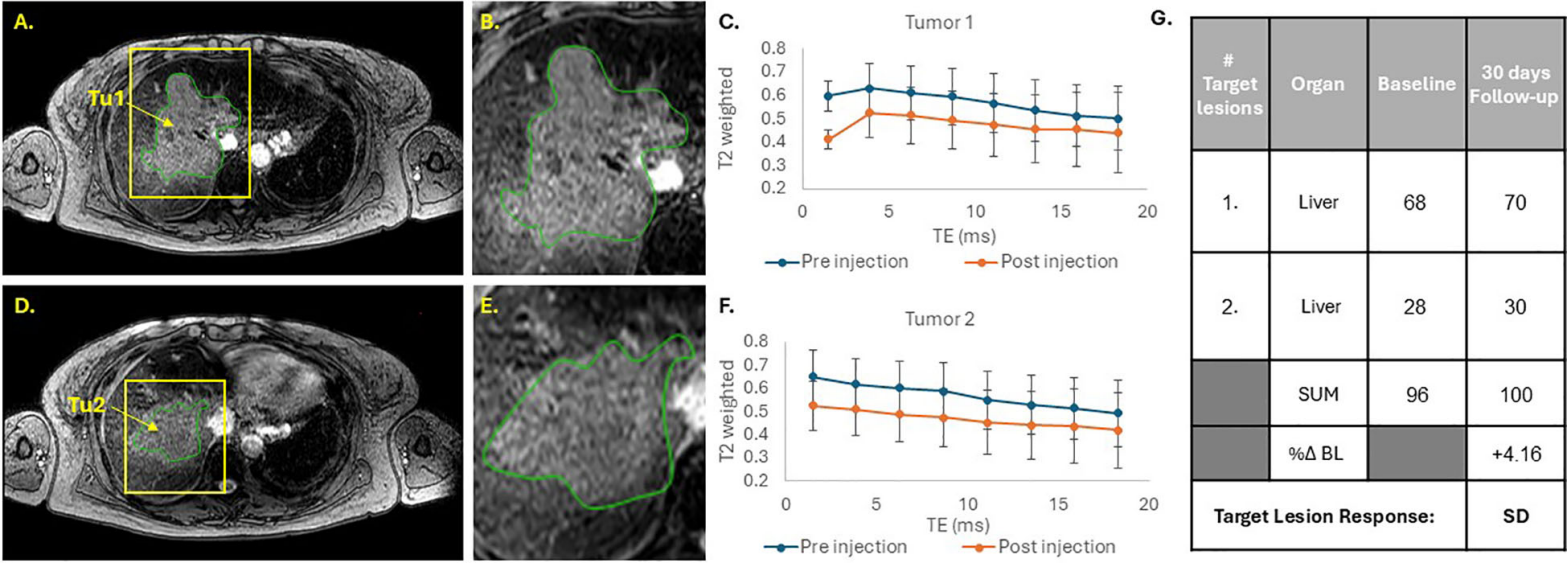
MRI analysis of SaNP accumulation in tumors of patient 2. T2*-weighted MR images **(A, B, D, E)**. Yellow arrows indicate tumor locations. Tumors are marked in green. T2*-weighed signals of tumor Tu1 **(C)** and tumor Tu2 **(F)**. Blue line – pre-injection (baseline); Orange line – post-injection (~4hrs.) and grey line – 30 days after treatment. Echo time (TE) was measured in milliseconds (ms). CT assessments and RECIST 1.1 evaluation **(G)**.

Two large heterogeneous liver tumors were identified and selected for analysis in patient 2, based on CT imaging. A slightly hyperdense hypermetabolic lesion was identified in the left liver lobe and marked as tumor 1 (Tu1), and an additional large heterogeneous lesion with hyperdense margins and a large central hypodense necrotic area was marked as tumor 2 (Tu2). Accumulation of the SaNP was visualized in both tumors (**Figure 3**) as areas of low signal intensity on T2*-weighted MR imaging. As with patient 1, CT imaging before treatment (baseline) and at the follow-up timepoint of 30 days post treatment in this patient revealed no significant changes in the tumor size (%Δ BL) which was only 4.16%, consistent with stable disease by RECIST 1.1.

Of note, in both patients, treatment with SaNP halted disease progression in contrast to previous drug therapies these patients received prior to enrolling in this trial where mild disease progression was present (3.2% per month for patient 1, and 10.5% for patient 2).

In terms of toxicities, both patients tolerated the procedure well without any evidence of significant laboratory abnormalities or any other toxicities beyond grade 2 (see all reported AEs in **Table 1**), other than mild localized and transient discomfort in the region of the lumbar spine which ceased immediately after the irradiation ended, as reported in the pain and discomfort questionnaire.

**Table 1:** Adverse events.

Adverse event	Patient 1	Patient 2	Total No. of AEs	Treatment-related AEs
Any adverse event	4	5	9	2
Back pain during SaNP administration	--	Grade 2		No
Loss of appetite	Grade 2	--	1	No
Lower back pain during AMF exposure	Grade 2	Grade 2	2	Yes
Heat sensation during AMF exposure	Grade 2	Grade 2	2	Yes
Rash	--	Grade 1	1	No
Vomiting	--	Grade 2	1	No
Worsening of fatigue	Grade 2	--	1	No

Grading of adverse events: Grade 1 – Mild symptoms causing no or minimal interference with usual social & functional activities with intervention not indicated; Grade 2 – Moderate symptoms causing greater than minimal interference with usual social & functional activities with intervention indicated; Grade 3 – Severe symptoms causing inability to perform usual social & functional activities with intervention or hospitalization indicated; Grade 4 – Potentially life-threatening symptoms.

The oral and surface temperatures of the patients were continuously monitored throughout the irradiation procedure. Oral temperatures were within the physiological range in both patients, measuring 37.1°C before irradiation and 36.8°C at the end of AMF exposure in patient 1 and 37.0°C before irradiation and 37.1°C in patient 2 at the end of AMF exposure, increasing in this patient by only 0.1°C. Surface temperature monitoring indicated stable readings in all probes. The heating rate as recorded by the probe located in the region of the lumbar spine was 0.8°C/min. after the first irradiation interval, 0.6°C/min. after the second interval, and 0.3°C/min. after the third interval in patient 1. In patient 2, a similar trend was observed using a probe located in the region of the upper lumbar spine. Any increase in the heating rate below 2°C/min. is considered non clinically significant. No other significant changes in temperature monitoring were observed. The changes in temperature during the treatment correlated well with the patients’ report of discomfort and heat sensation in these areas.

## Discussion

Preliminary analysis of two patients with advanced primary liver cancer who failed several lines of drug therapy and were treated with magnetic nanoparticle-based hyperthermia demonstrated the ability of treatment to stabilize disease progression without causing any toxicities. As such, this new therapy could prove useful in stabilizing disease progression in patients with advanced cancer especially given the lack of toxicities. Indeed, our two patients tolerated the procedures well without incurring any meaningful or significant side effects during treatment other than minor feeling of heat in the lumbar spine. Although the data is preliminary, if confirmed at the interim and final analyses of the trial, this non-invasive targeted nanotechnology could prove to be care changing as patients who fail traditional therapies have limited viable options. A particularly attractive aspect of this novel approach which delivers large amounts of ferromagnetic nanoparticles to tumors, in order to selectively heat them without damaging healthy tissues, is its ability to be imaged during and after treatment to monitor the delivery to and subsequent accumulation of the nanoparticles in the tumors by using MR sequences that are sensitive to ferromagnetic particles. Such proof-of-concept was clearly established with these two patients as the delivery and subsequent clearance of the nanoparticles was indeed visualized on T2*-weighted MR imaging thereby providing the necessary confirmation of adequate delivery to the target tumors and at the same time of its clearance over time via the liver and spleen. It is also interesting to note that the tumors that accumulated the greatest amounts of nanoparticles were the ones that also responded as assessed by RECIST 1.1.

As mentioned above, the treatment also had a good safety profile without significant AEs. The two patients only complained of some discomfort in the area of their lumbar spine due to the generation of hotspots during AMF exposure which was predicted by a computational human anatomical model (12) and observed in other patients treated to date (6, 7). These hotspots are a direct result of eddy currents, induced when exposed to a time-varying magnetic field (13). Yet, the use of a cooling blanket has proven effective, enabling control of surface temperature and preventing undesired heating of tissues resulting from eddy currents. Temperature monitoring during AMF irradiation confirmed the absence of significant changes in either core or surface body temperatures. An improvement introduced in the treatment of these two patients included using a pulsed AMF irradiation mode, rather than irradiation with a constant field as had previously been done. A pulsed AMF can reduce unwanted eddy currents and associated heating in tissues by using a specific duty cycle. This approach allows for brief pulsed AMF exposure, enabling heat dissipation from the tissue before the next exposure, effectively lowering the cumulative heating of healthy tissues (14). Our findings indicate that this approach is indeed safe and effective.

In conclusion, our preliminary results indicate that treatment with SaNP followed by pulsed AMF irradiation had encouraging results in patients with advanced primary liver cancer by stabilizing disease progression in heavily pre-treated patients, without causing any high-grade toxicities or side effects.

## Data Availability

The study is an ongoing First-in-Human clinical trial. Requests to access the datasets should be directed to Sarahk@newphase.co.il.

## Glossary

AEs: Adverse Events
AMF: Alternating Magnetic Field
CT: Computed Tomography
CTCAE: Common Terminology Criteria for Adverse Events
ECOG: Eastern Cooperative Oncology Group
EPR: Enhanced Permeability and Retention
HCC: Hepatocellular Carcinoma
IO: Iron Oxide
MRI: Magnetic Resonance Imaging
RECIST: Response Evaluation Criteria in Solid Tumors
SaNP: Sarah Nanoparticle
SAEs: Serious Adverse Events
